# Association between *Platelet-Derived Growth Factor Receptor Alpha* Gene Polymorphisms and Platelet-Rich Plasma’s Efficiency in Treating Lateral Elbow Tendinopathy—A Prospective Cohort Study

**DOI:** 10.3390/ijms25084266

**Published:** 2024-04-12

**Authors:** Alicja Jarosz, Anna Balcerzyk-Matić, Joanna Iwanicka, Tomasz Iwanicki, Tomasz Nowak, Karol Szyluk, Marcin Kalita, Sylwia Górczyńska-Kosiorz, Wojciech Kania, Paweł Niemiec

**Affiliations:** 1 Department of Biochemistry and Medical Genetics, School of Health Sciences in Katowice, Medical University of Silesia in Katowice, Medykow 18 Str., 40-752 Katowice, Poland; alicja.jarosz@sum.edu.pl (A.J.); abalcerzyk@sum.edu.pl (A.B.-M.); jiwanicka@sum.edu.pl (J.I.); tiwanicki@sum.edu.pl (T.I.); tnowak@sum.edu.pl (T.N.); 2 District Hospital of Orthopaedics and Trauma Surgery, Bytomska 62 Str., 41-940 Piekary Sląskie, Poland; karol.szyluk@sum.edu.pl (K.S.); marcin.kalita1991@gmail.com (M.K.); 3 Department of Physiotherapy, Faculty of Health Sciences in Katowice, Medical University of Silesia in Katowice, Medykow 12 Str., 40-752 Katowice, Poland; 4 Department of Internal Medicine, Diabetology and Nephrology, School of Medicine with the Division of Dentistry in Zabrze, Medical University of Silesia in Katowice, 41-800 Zabrze, Poland; skosiorz@sum.edu.pl; 5 Department of Trauma and Orthopedic Surgery, Multidisciplinary Hospital in Jaworzno, Chełmońskiego 28 Str., 43-600 Jaworzno, Poland; wojtekkania@poczta.onet.pl

**Keywords:** platelet-rich plasma, PRP, platelet-derived growth factor receptor alpha, *PDGFRA*, lateral elbow tendinopathy, tennis elbow, single nucleotide polymorphisms, SNP

## Abstract

Individual differences in the response to platelet-rich plasma (PRP) therapy can be observed among patients. The genetic background may be the cause of this variability. The current study focused on the impact of genetic variants on the effectiveness of PRP. The aim of the present study was to analyze the impact of single nucleotide polymorphisms (SNP) of the platelet-derived growth factor receptor alpha (*PDGFRA*) gene on the effectiveness of treating lateral elbow tendinopathy (LET) with PRP. The treatment’s efficacy was analyzed over time (2, 4, 8, 12, 24, 52 and 104 weeks after the PRP injection) on 107 patients using patient-reported outcome measures (PROM) and achievement of a minimal clinically important difference (MCID). Four SNPs of the *PDGFRA* gene (rs7668190, rs6554164, rs869978 and rs1316926) were genotyped using the TaqMan assay method. Patients with the AA genotypes of the rs7668190 and the rs1316926 polymorphisms, as well as carriers of the T allele of rs6554164 showed greater effectiveness of PRP therapy than carriers of other genotypes. Moreover, the studied SNPs influenced the platelets’ parameters both in whole blood and in PRP. These results showed that *PDGFRA* gene polymorphisms affect the effectiveness of PRP treatment. Genotyping the rs6554164 and the rs1316926 SNPs may be considered for use in individualized patient selection for PRP therapy.

## 1. Introduction

Receptor tyrosine kinases (RTK) are transmembrane proteins that are activated by growth factors. Their activation leads to cells’ migration, proliferation and/or differentiation. The platelet-derived growth factor receptor (PDGFR) family includes RTKs that regulate various cellular activities [1]. PDGFRs are constructed from two monomers, PDGFRα and PDGFRβ, which are encoded, respectively, by the platelet-derived growth factor receptor alpha (*PDGFRA*) and platelet-derived growth factor receptor beta (*PDGFRB*) genes. These monomers form PDGFRα, PDGFRβ homodimers and a PDGFRα/β heterodimer [2]. Dimerization of two PDGFRs occurs after binding with one of the platelet-derived growth factor (PDGF) ligands, also resulting in the formation of homo- or heterodimers [1]. The PDGFRα receptor binds all PDGF chains except PDGF-D, whereas PDGFRβ binds the PDGF-B and PDGF-D peptides. Thus the PDGFRα homodimer binds most of the PDGF isoforms (PDGF-AA, PDGF-AB, PDGF-BB and PDGF-CC) [3]. PDGFR homodimers regulate cell differentiation and proliferation pathways, but each homodimer is also responsible for regulating unique signaling pathways. Furthermore, some signaling is solely modulated through the heterodimeric PDGFRα/β type of receptor [4].

PDGFRs and PDGFs play important roles in tissue regeneration. PDGFRs are expressed by several cell types involved in wound healing, such as fibroblasts, smooth muscle cells, neutrophils and macrophages. PDGFs released from platelets can recruit these cells to the wounded areas [5]. Moreover, PDGFs stimulate the macrophages to produce and secrete other growth factors that are associated with the healing process [2]. PDGFs can also stimulate the proliferation of fibroblasts, the production of collagen [5] and the activation of matrix metallopeptidase 1 (MMP-1), which takes part in tissue remodeling [2]. However, it should be mentioned that although these processes are involved in regeneration, they may also contribute to the development of tendinopathy [6]. For this reason, the activity of growth factors must be precisely regulated for proper regeneration, which is also influenced by the activity of their receptors.

Lateral elbow tendinopathy (LET), also known as tennis elbow, is a degenerative condition of the proximal attachment of the extensor muscles to the humeral epicondyle [7]. In recent years, a popular option for treating LET is by the injection on platelet-rich plasma (PRP) [8]. PRP is the processed liquid fraction of autologous peripheral blood with a platelet concentration above the baseline and a high concentration of growth factors [9]. The effectiveness of PRP in treating musculoskeletal injuries is not consistent, leading to controversy regarding its usage in therapy [10]. Since growth factors are responsible for the regenerative properties of PRP, we decided to analyze whether single nucleotide polymorphisms (SNPs) of the genes encoding these growth factors and their receptors affect the effectiveness of PRP therapy. The current study is a part of a larger series of works focusing on the impact of genetic variants on the effectiveness of PRP therapy in lateral elbow tendinopathy. Due to the complexity of the pathogenesis of tendinopathy, there are numerous candidate genes whose genetic variability may modify the efficacy of treatment. So far, we have examined the *PDGFA* [11], *PDGFB* [12], *PDGFRB* [13] and vascular endothelial growth factor (*VEGFA*) [14] genes. In the current study, we added the *PDGFRA* gene to the previously analyzed gene panel. In order to analyze the impact of the studied polymorphisms on the effectiveness of the therapy, we checked their association with patient-reported outcome measures (PROM), namely the visual analog scale (VAS); the quick version of the disabilities of the arm, shoulder and hand (QDASH); the patient-rated tennis elbow evaluation (PRTEE) and achievement of a minimal clinically important difference (MCID).

## 2. Results

### 2.1. Characteristic of the PDGFRA Gene’s Polymorphisms

**Figure 1:**
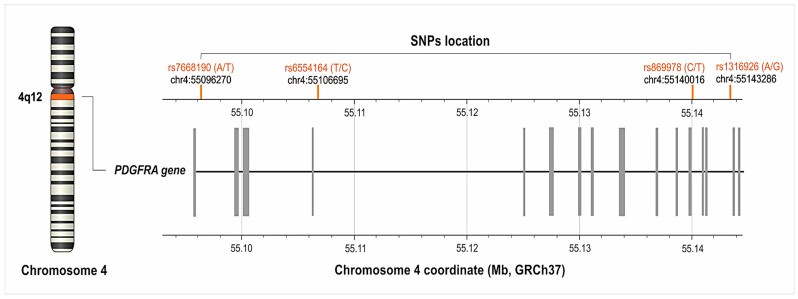
Location of the *PDGFRA* gene’s single nucleotide polymorphisms (SNPs) on chromosome 4 (GRCh37.p13 chr 4). The figure was created on the basis of data from LDmatrix Tool [16].

We analyzed four different SNPs of *PDGFRA*: rs7668190 (A>T), rs6554164 (T>C), rs869978 (T>C) and rs1316926 (G>A). The frequency of their alleles and genotypes, along with their chromosomes and gene locations, is described in Table 1 and Figure 1. The genotype distribution of individual SNPs was consistent with Hardy–Weinberg equilibrium (*p* > 0.050).

**Table 1:** Alleles, genotypes and frequency of the studied polymorphisms of the *PDGFRA* gene. Chromosome 4 coordinates, the *PDGFRA* gene coordinates and the minor allele frequency are based on the database of SNPs of the National Center for Biotechnology Information, U.S. National Library of Medicine [15].

SNP	Chromosome 4 Coordinate *	*PDGFRA* Gene Coordinate	Minor Allele	Minor Allele Frequency (%) **	Alleles	*n*	(%)	Genotype	*n*	(%)
rs7668190	55096270	6007	T	27.63	A	118	(62.43)	AA	61	(46.21)
					T	71	(37.57)	AT	57	(43.18)
								TT	14	(10.61)
								AA + AT	118	(89.39)
								TT + AT	71	(53.79)
rs6554164	55106695	16432	C	22.96	T	124	(69.27)	TT	76	(58.02)
					C	55	(30.73)	CT	48	(36.64)
								CC	7	(5.34)
								TT + CT	124	(94.67)
								CC + TT	55	(41.98)
rs869978	55140016	49753	T	22.17	C	127	(74.71)	CC	89	(67.42)
					T	43	(25.29)	CT	38	(28.79)
								TT	5	(3.79)
								CC + CT	127	(96.21)
								TT + CT	43	(32.58)
rs1316926	55143286	53023	G	48.61	A	108	(55.10)	AA	44	(33.33)
					G	88	(44.90)	AG	64	(48.48)
								GG	24	(18.18)
								AA + AG	108	(96.97)
								GG + AG	88	(66.67)

Legend: SNP, single nucleotide polymorphism. * GRCh37.p13 chr 4, ** 1000 Genomes, Europe.

**Figure 2:**
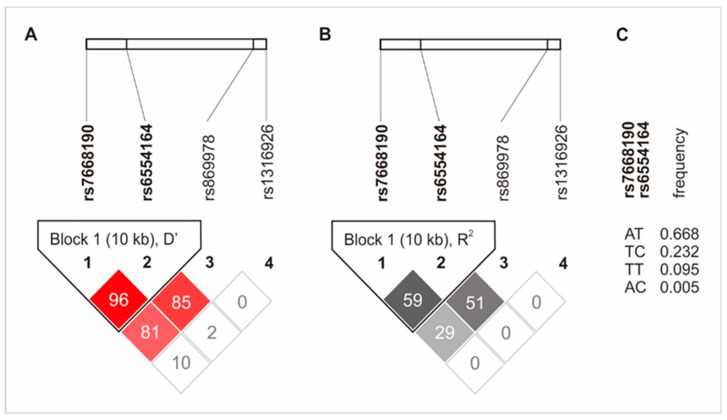
Haplotype analysis of the *PDGFRA* gene’s polymorphisms. Results for the D’ (**A**), R^2^ (**B**) and frequency of haplotypes (**C**). The colors highlight the degree of linkage disequilibrium between SNPs. The darker the color, the greater D’ or R2.

The rs7668190 and rs6554164 polymorphisms were linked and formed a haplotype block (10 kb). The rs869978 SNP was also linked to the rs6554164 and the rs7668190 SNPs, but to a lesser extent. The linkage disequilibrium between the studied polymorphisms is shown in Figure 2.

### 2.2. The Influence of the Studied Polymorphisms on PROM Values, Achievement of MCID and Platelet Parameters

**Figure 3:**
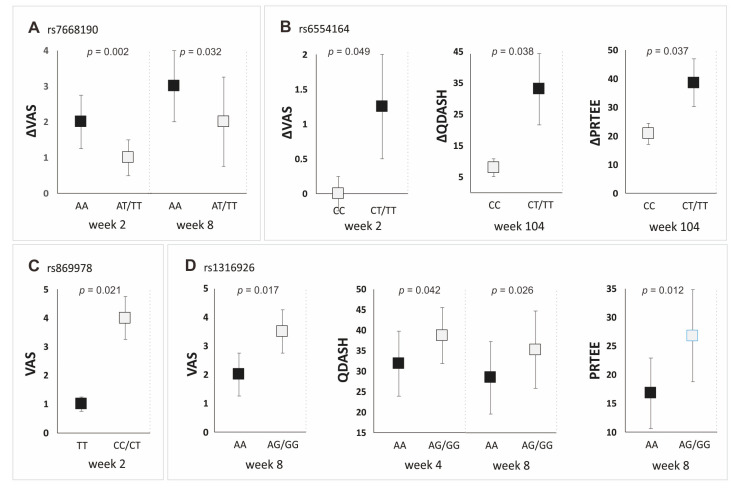
Median (±QD) values of patient-reported outcome measures for genotypes of the *PDGFRA* gene’s polymorphisms (dominant/recessive model). Results for the rs7668190 (**A**), rs6554164 (**B**), rs869978 (**C**), and rs1316926 (**D**) polymorphisms. Legend: VAS, visual analog scale; QDASH, quick version of disabilities of the arm, shoulder and hand; PRTEE, patient-rated tennis elbow evaluation.

All of the analyzed SNPs showed statistically important associations with PRP’s effectiveness, although to varying degrees (Figure 3). Patients with the AA genotype (rs7668190) had higher values of ΔVAS at Weeks 2 and 8 of follow-up. The carrier status of the allele T (rs6554164) was associated with higher values of ΔVAS (at Week 2), ΔQDASH (at Week 104) and ΔPRTEE (at Week 104 of follow-up). Carriers of the AA genotype (rs1316926) had lower VAS (at Week 8), QDASH (at Weeks 4 and 8) and PRTEE (Week 8) values than G allele carriers. The TT homozygosity of the rs869978 SNP was also associated with better effectiveness of PRP therapy (lower VAS at Week 2). Detailed results for all follow-up points are presented in the Supplementary Tables (Tables S1–S4).

Moreover, the studied polymorphisms influenced the achievement of a minimal clinically important difference in terms of VAS and QDASH, both in the additive and dominant/recessive models (Table 2). Patients with the AA genotype of the rs1316926 and the rs7668190 polymorphisms achieved MCID more often than the carriers of other genotypes. In the case of the rs6554164 polymorphism, the achievement of MCID was more common for patients with the TT genotype in the additive model, and carriers of the T allele in the dominant/recessive model (Table 2).

The analyzed SNPs were also associated with the platelets’ parameters. The impact of genotypes associated with higher PRP efficacy on platelets varies, depending on the specific polymorphism. Carriers of the AA genotype of the rs7668190 SNP had higher platelets (PLT) and plateletcrit (PCT) in the PRP, while carriers of the AA genotype of the rs1316926 SNP had lower PLT in the PRP. In the case of the rs869978 SNP, patients with the TT genotype were characterized by lower PLT and PCT as well as a higher mean platelet volume (MPV) and platelet distribution width (PDW) in whole blood. The association of the studied SNPs with the platelets’ parameters is shown in Table 3. Detailed results for whole blood and PRP parameters are presented in the Supplementary Table (Table S5).

## 3. Discussion

The present study focused on analyzing the influence of the *PDGFRA* gene’s polymorphisms on the effectiveness of PRP therapy in treating LET. According to our results, all the studied SNPs were associated with PRP’s efficacy; however, the level of influence varied between specific polymorphisms. The greatest impact was shown for the rs6554164 and the rs1316926 SNPs. One of those SNPs, rs6554164, formed a haplotype block with rs7668190. The most frequent haplotype, AT (rs7668190 and rs6554164, respectively), contained alleles associated with more successful therapy from both SNPs. Moreover, three of four analyzed polymorphisms were associated with achievement of MCID. The MCID is used to determine whether significant progress has been made in a patient’s therapy [17]. The association of these SNPs with MCID further demonstrated their influence on the treatment’s effectiveness and highlights their impact on the course of therapy from the patient’s perspective. In addition, the studied polymorphisms (except for rs6554164) influenced the platelets’ parameters. However, the effect of these SNPs on platelets is not consistent, as the genotypes associated with higher PRP efficacy of the rs7668190 polymorphism were associated with higher PLT and the rs1316926 was associated with lower PLT in PRP.

PDGF receptors (both PDGFRα and PDGFRβ) are expressed in tenocytes and have an important role in the remodeling of tendon tissue [18,19,20]. The PDGFRα receptor is expressed in most tendon fibroblasts [18]. Interestingly, expression of the *PDGFRA* gene was shown in tenocytes obtained from both healthy and diseased human tendons [19]. PDGFR-blocked tendons showed deficits in the synthesis and remodeling of extracellular matrixas well as cell differentiation and proliferation. Furthermore, inhibition of PDGFR prevents the growth of tendon tissue after mechanical overload [18]. Due to the fact that LET and other tendinopathies are caused by mechanical overload, PDGFRα’s activity seems to be very important in the treatment of these conditions. The effect of PDGFRα on tendon regeneration has also been demonstrated in studies of murine potential tendon stem cells and tubulin polymerization-promoting protein family member 3-expressing cells (*Tppp3+*). These cells can generate new tenocytes and self-renew after damage. Some *Tppp3+* cells showed the expression of PDGFRα. PDGF-AA induces the production of new tenocytes by *Tppp3+* cells, while the inactivation of PDGFRα in *Tppp3+* cells blocks tendons’ regeneration. Moreover, PDGFRα signaling is necessary and sufficient for the *Tppp3+* cell line to generate new tenocytes. Interestingly, *Tppp3+ Pdgfra+* cells did not participate in the formation of tenocytes during homeostasis, suggesting that they are activated only after injury [20]. In turn, studies on murine cells expressing stage-specific embryonic antigen 4 (*SSEA-4*), cluster of differentiation 90 (*CD90*) and *PDGFRA*(*Ssea-4+ Cd90+ Pdgfra+* subpopulation) isolated directly from the adipose tissue fraction showed a high potential for proliferation and differentiation, especially towards tendon tissue [21]. This further demonstrates the importance of the PDGFRα receptor in the proliferation of tendon cells and the regeneration of tendon tissue.

PDGFs and PDGFRs, including PDGFRα, also participate in angiogenesis [22,23,24]. Most literature about PDGFRα’s role in angiogenesis is related to cancer research [22,23,25]. PDGFs participate in the proliferation, angiogenesis, migration, and invasion of many tumors; therefore, targeting the PDGF/PDGFR signaling pathway is being considered for cancer therapy [22,23]. Nevertheless, the course of angiogenesis also has a major role during LET treatment. Proper angiogenesis is necessary during wound healing and regeneration; however, pathological neovascularization is one of the main characteristics of tendinopathy [26]. Research has shown that the PDGFRα homodimer promotes angiogenesis after binding with PDGF-AA and PDGF-CC [22]. PDGFRα knockout mice showed significant vascular abnormalities [24]. Pro-angiogenic PDGFs also have a significant regulatory role in the development of healthy or unhealthy blood vessels [23]. Moreover, PDGFs can indirectly stimulate angiogenesis by increasing the expression of VEGF, a crucial protein during neovascularization [25]. PDGFRα’s role in angiogenesis was also shown in patients with Moyamoya disease, in which PDGFRα signals play an important role in developing spontaneous angiogenesis between the temporal muscle and the neocortex [27]. There are no studies in the literature assessing the effect of PDGFRα on angiogenesis in the course of tendinopathy, although it was shown that PDGFR-blocked tendons show deficits in neovascularization [18]. It can be assumed, on the basis of other studies, that the PDGFRα receptor is involved in the course of angiogenesis during tendinopathy, which may affect the development and treatment of this condition. Nevertheless, further research is necessary to precisely determine the role of PDGFRα in this regard.

PDGFRα may also affect the effectiveness of PRP itself by influencing platelets’ activity. Human platelets have functionally active PDGFRα receptors but not PDGFRβ [28,29,30]. Moreover, PDGFRα is involved in negative feedback regulation during the activation of platelets. Platelets secrete PDGFs that bind with PDGFRα receptors, which leads to a reduction in the platelets’ activity [28]. This mechanism probably limits the excessive activity of platelets. Moreover, according to our results, the SNPs of the *PDGFRA* gene are associated with the platelets’ parameters both in whole blood and in PRP. The rs7668190 SNP was associated with PLT and PCT in PRP, the rs1316926 SNP was associated with PLT in PRP, and the rs869978 SNP was associated with all the platelets’ parameters in whole blood, i.e., PLT, PCT, MPV and PDW. It seems obvious that the number of platelets will affect PRP’s effectiveness, but our results suggest that this dependence is not so simple. It is worth emphasizing that the volume of platelets is associated with their function and therefore can influence PRP’s efficacy as well. Increased MPV correlates with higher platelet activity [31,32,33]. Larger platelets have higher protein levels [34,35] as well as greater amounts of α-granules and growth factors [36]. Additionally, according to Ozer et al. [37], MPV should be used for the standardization of PRP due to its impact on platelets’ activity, and the fact that specific layers of centrifuged plasma have different levels of MPV. Importantly an increase in MPV is usually associated with a decrease in the platelet count [38,39,40]. This is due to the principles of platelets’ physiology. The body maintains the mass of platelets, not the platelet count [41]. That explains why genotypes related to greater PRP efficacy were also associated with lower PLT and higher MPV in whole blood (rs869978), as well as lower PLT in PRP (rs1316926). These results show that PRP’s effectiveness is mostly influenced by platelets’ activity and the balance between PLT and MPV. This may also be a reason why the genotype of the rs7668190 polymorphism related to the greater PRP efficacy of the rs7668190 SNP is associated with higher PLT in PRP. Patients with the TT genotype of the rs7668190 polymorphism had a median platelet concentration in PRP of 286.50 × 109/L, which appears to be low compared with the homozygous AA genotype of the same polymorphism, where the median platelet concentration in PRP was 349.00 × 109/L (Table S5). Interestingly, such low platelet concentrations in PRP were not observed in the case of other genotypes of the *PDGFRA* gene, except for the CC homozygosity of the rs6554164 polymorphism, which, similar to the TT homozygosity (rs7668190), is characterized by lower effectiveness of PRP therapy, although the differences in the platelet concentration within the variants of rs6554164 polymorphism did not show statistical significance. It is possible that in the case of rs7668190 and rs6554164 polymorphisms, the lower level of PLT in PRP (visible in patients with the TT and CC genotypes, respectively) is too low to ensure the proper functioning of PRP, which leads to the opposite results for these SNPs than for others. It is likely that the PLT levels in PRP must be within the proper range for PRP therapy to be effective. Too high a PLT level is associated with the presence of a higher number of smaller and less active platelets, and too low a PLT level is associated with the presence of more active platelets, but in small amounts, which, in both cases, may lead to a reduction in the effectiveness of PRP treatment. However, this issue requires more research and thorough examination.

Out of all the PDGF receptors, the PDGFRα homodimer binds most of the PDGF growth factors and is responsible for most signaling pathways. Activation of the PDGFRα homodimer leads to cells’ proliferation, migration and differentiation, as well as angiogenesis and tissue healing [22]. All of these processes are important during the treatment of tendinopathy. During tendinopathy, the extracellular matrix is disturbed due to abnormalities in the ratio of Collagen I to Collagen III [6], the balance between matrix metallopeptidases (MMPs) and tissue inhibitors of metalloproteinases (TIMPs) is lost [42], and hypervascularization occurs [43]. Currently, failed healing is considered one of the most likely causes of tendinopathy [44]. It leads to disruptions in the structure of collagen, inflammation, disturbances in angiogenesis and the disordered proliferation of tenocytes [6]. Therefore, it seems that the same processes that are involved in the treatment of tendinopathy also contribute to its development. The therapeutic effects of PRP in treating tendinopathy include stimulating cell proliferation, the synthesis of collagen, the regulation of angiogenesis, and modulation of inflammation [26]. The growth factors contained in PRP, including PDGFs, are responsible for the PRP’s properties. As mentioned above, PDGFRα contributes to the proliferation of tenocytes [6], modulation of angiogenesis [22] and the regulation of platelets’ activity [28], while PDGFs are involved in the synthesis of collagen [5] and activation of MMPs [2]. For these reasons, PDGFRα appears to be strongly involved in the development of tendinopathy, its treatment and the effects of PRP. It is also worth emphasizing that all the above mechanisms must be appropriately regulated to ensure therapeutic success. Therefore, the proper functioning of PDGFRα seems to be important in the treatment of LET with PRP, which was confirmed by our results.

The main limitation of this research is the relatively small study group. Hence, to confirm the obtained results, similar analyses on a larger group are recommended. Another important limitation is the inclusion of patients using additional forms of therapy (physiotherapy and non-steroidal anti-inflammatory drugs) after administration of PRP. It is worth emphasizing, however, that no statistically significant differences were found in the frequency of additional therapy among the carriers of the respective *PDGFRA* genotypes. We considered it unethical to deny patients access to other forms of treatment if PRP therapy was ineffective. The advantages of the study are the long follow-up (2 years after PRP treatment), the precise quantitative (PROM) and qualitative (MCID) analyses, and the clinical and ethnic homogeneity of the study group. In summarizing the limiting factors, it is also important to emphasize the significant interpretational challenge arising from the lack of any literature data on the studied polymorphisms of the *PDGFRA* gene. Two of them, namely rs7668190 and rs6554164, were analyzed within genome-wide association studies (GWAS) of traits such as BMI, height, waist–hip ratio, the parameters of carbohydrate and hormonal metabolism, astigmatism and pulmonary function [45]. However, in each of these studies, the statistical significance of the differences did not exceed the threshold for GWAS studies. Therefore, the potential biological role of these polymorphisms, for example as markers of traits, will remain unknown without additional research.

In conclusion, we showed that the rs7668190, rs6554164, rs869978 and rs1316926 polymorphisms of the *PDGFRA* gene are associated with PRP’s effectiveness. The greatest impact on the effect of PRP was from the rs6554164 and the rs1316926, an impact which was shown both in quantitative (PROM) and qualitative (MCID) analyses. Moreover, the rs6554164 SNP formed a haplotype with the rs7668190 SNP. Furthermore, the studied polymorphisms influenced the platelets’ parameters, although the results in this regard were not consistent. This study provided new data on the factors influencing PRP therapy. However, in order to fully understand the involvement of PDGFRα in the development of tendinopathy, its treatment and its effects on platelets, further research is necessary.

## 4. Materials and Methods

This study adhered to the STROBE and MIBO guidelines. The study protocol received approval from the Ethics Committee of the Medical University of Silesia in Katowice, Poland (KNW/0022/KB1/24/I/17). Our research methods adhered to the Helsinki Declaration of 1975 and its subsequent revisions, ensuring that ethical standards were met. All participants provided informed written consent.

This study used the same measures of effectiveness, follow-up timeline, patient selection method, injection of PRP and blood analyses as our prior studies [11,12,13,14]. The research cohort consisted of patients diagnosed with lateral elbow tendinopathy who underwent PRP treatment. Their progress was examined over a span of 2 years at 2, 4, 8, 12, 24, 52 and 104 weeks after the PRP injections using PROMs, namely the VAS, QDASH and PRTEE. Outcome values were compared with each patient’s clinical condition on the day of the injection (baseline at 0 weeks). The VAS, QDASH and PRTEE questionnaires were utilized to assess pain and disability. The VAS scores ranged from 0 (minimum pain) to 10 (maximum pain), while the QDASH and PRTEE scores ranged from 0 (minimum pain and disability) to 100 (maximum pain and disability). We used a translation and cultural adaptation of the Polish version of the QDASH [46] and PRTEE questionnaires [47]. Moreover, we assessed the MCID for each PROM at each follow-up point. The achievement or failure to achieve an MCID was determined on the basis of the literature and weighted: 1.5 points for VAS [48], 15.8 points for DASH [49] and 11 points for PRTEE [50]. Patients that achieved MCID were specified as the MCID+ group, whereas patients who did not achieve MCID were classed as MCID–.

### 4.1. Patient Selection

Patient selection occurred between November 2018 and November 2019, and data collection continued until November 2020. The study encompassed 107 Polish Caucasians residing in Upper Silesia, including 65 females and 42 males aged 24 to 64 years, all diagnosed with LET (coded as M77.1 in the International Statistical Classification of Diseases and Related Health Problems, 10th Revision, ICD-10). All patients exhibited typical symptoms of LET, including pain around the common extensor’s origin, tenderness at palpation over the lateral epicondyle of the humerus, muscle weakness, morning stiffness, a history of limb overuse or injury and positive results for Thomson’s test, Mill’s test and Cozen’s sign. These patients received autologous platelet-rich plasma treatment at either the VI Department of Trauma and Orthopedics, District Hospital of Orthopedics and Trauma Surgery in Piekary Sląskie, Poland, or the Department of Orthopedic Trauma Surgery, Multidisciplinary Hospital in Jaworzno, Poland. All patients were chosen, examined and treated by the same orthopedic surgeons (K.S. in Piekary Sląskie and W.K. in Jaworzno), following an identical study protocol.

**Figure 4:**
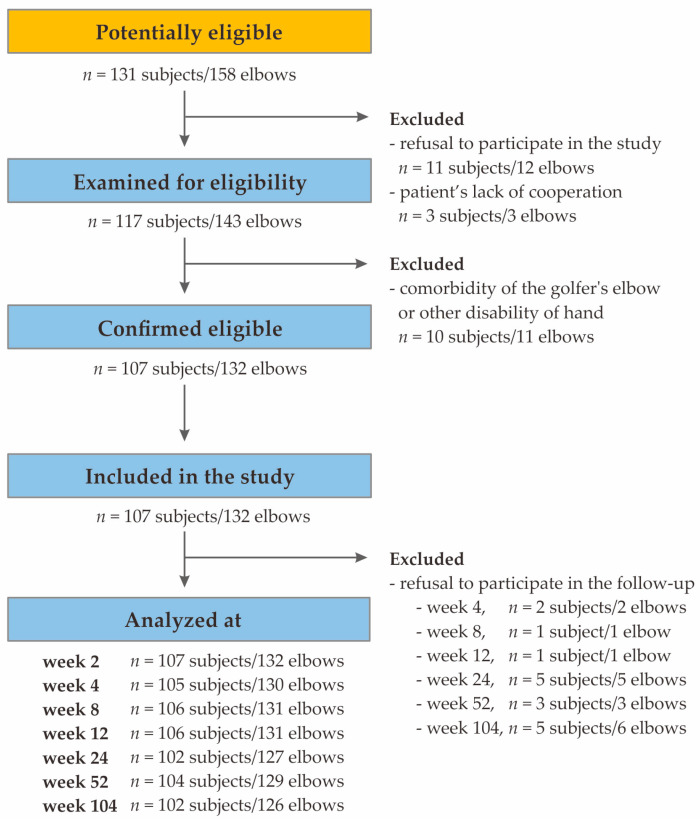
Flowchart of the study’s selection process.

Exclusion criteria encompassed additional injuries or diseases (e.g., rheumatoid arthritis, active malignancies, cervical radiculopathy), previous PRP injections, prior surgical procedures, local steroid injections within the preceding 6 months, anti-platelet medications or pregnancy. Notably, there was no specific post-injection rehabilitation protocol in this study. Additional post-injection therapies, including steroids, non-steroidal anti-inflammatory drugs, physiotherapy, and additional PRP injections were monitored but were not used as exclusion criteria. The flow diagram depicting the patients included in the study is provided in Figure 4.

### 4.2. Characteristics of the Study Group 

The study group comprised 107 patients, including 132 elbows with 25 bilateral cases. There were 65 females and 42 males, aged 24–64 years (median ± QD: 46.00 ± 5.50). The prevalent comorbidities included hypertension, thyroid disease and gout. The mean white blood cell (WBC) concentration was 6.26 ± 1.16 (109/L ± QD), the PLT level was 240.00 ± 40.50 (109/L ± QD) while the MPV was 9.10 ± 0.73 (fL ± QD). Females exhibited higher platelet levels (261.50 ± 33.00 vs. 224.00 ± 38.75, respectively, *p* = 0.000) and plateletcrit (2.37 ± 0.36 vs. 2.04 ± 0.33, respectively, *p* = 0.001) in whole blood compared with males. The platelets’ parameters in PRP showed no variance between the sexes. An overview of the clinical details is shown in Table 4.

### 4.3. PRP Separation, Injection Procedure, Whole Blood and PRP Parameters

Blood was collected under standardized conditions in a treatment room equipped with disposable materials and maintained at 20 °C under consistent lighting conditions. An Arthrex Autologous Conditioned Plasma double syringe from Arthrex GmbH, München, Germany, was used for extraction of the plasma. PRP was separated from fresh whole blood immediately after collection. From each patient, 12 mL of whole blood was collected using a 1.2 mm needle, mixed with 3.13% sodium citrate (MediPac^®^ GmbH, Königswinter, Germany) in a 9:1 ratio and then centrifuged under the same conditions using a Rotofix 32A centrifuge (Andreas Hettich GmbH & Co., Tuttlingen, Germany) at a speed of 1500 rpm for 5 min. PRP was isolated twice (separately for each elbow) for patients with bilateral lateral elbow tendinopathy. After centrifugation, 2.5 to 3.5 mL of PRP was obtained from each blood sample. Fresh PRP, 2.0–3.0 mL in volume, was immediately injected into the region of the common extensor origin using a 1.2 mm needle under ultrasound control with the Mindray DC-3 apparatus (Mindray Medical Poland Sp. z o.o., Warsaw, Poland) equipped with a linear probe with a frequency range of 5, 7.5 and 10 MHz. The remaining 0.5 mL of PRP was reserved for further analysis.

After the injection of PRP, each patient was observed for 30 min to monitor potential complications, with particular attention to local inflammation and allergic reactions. Patients were discharged if there were no disturbing local or general symptoms and instructed to contact the hospital if they experienced side effects such as local inflammatory reactions or persistent pain. They were also advised to limit heavy use of the affected limb for 24 h. Notably, no patients developed infections at the PRP injection site.

On the day of the PRP injection, a complete blood count and hsCRP levels were determined in whole blood. Additionally, platelet-related measures, including platelets, PCT, MPV and PDW, were assessed in fresh PRP. For patients with bilateral tennis elbow receiving injections on the same day, a single sample of both whole blood and PRP was analyzed. If the injections were conducted on different dates, two separate tests of both whole blood and PRP were performed.

### 4.4. Genetic Analyses

Genomic DNA was isolated from peripheral blood leukocytes using the MasterPure genomic DNA purification kit from Epicenter Technologies in Madison, WI, USA. SNPs of the *PDGFRA* gene were genotyped using TaqMan Predesigned SNP Genotyping Assay kits and the 7300 Real-Time PCR System from Thermo Fisher Scientific in Waltham, MA, USA. The accuracy of genotyping was confirmed by regenotyping 10–15% of the samples, with 100% repeatability in the results. Genotypic data were obtained for 107 patients. The exception was the rs6554164 polymorphism, for which genotyping was unsuccessful in one individual.

Only SNPs with a MAF (minor allele frequency) of 20% or greater in populations of European origin, according to the database of SNPs of the National Center for Biotechnology Information, U.S. National Library of Medicine (accessed on 15 February 2023) [15], were selected for analysis. These included the variants rs7668190 (A>T), rs6554164 (T>C), rs869978 (T>C) and rs1316926 (G>A). All of studied variants are intronic polymorphisms. The location of the analyzed SNPs is shown in Figure 4.

### 4.5. Statistical Analysis

The impact of various quantitative and qualitative variables (such as age, sex, BMI, blood count, PRP parameters, *PDGFRA* genotypes, etc.) on the treatment’s effectiveness was assessed, accounting for the raw VAS, QDASH and PRTEE values; the follow-up values compared with the baseline (ΔVAS, ΔQDASH and ΔPRTEE) and the achievement of MCID.

Statistical analyses were conducted utilizing Statistica 13.0 software (TIBCO Software Inc., Palo Alto, CA, USA). The normality of the data’s distribution was assessed using the Shapiro–Wilk test. Since the quantitative variables displayed non-normal distributions, the Mann–Whitney U test was used for comparisons. Quantitative data were presented as the median with the interquartile range (QD). Qualitative data comparisons and Hardy–Weinberg equilibrium was assessed using the *χ*^2^ test. Fisher’s correction was applied to subgroups with fewer than 10 patients.

The genotype frequencies of the studied SNPs were consistent with Hardy–Weinberg equilibrium. Genetic data were examined under the dominant/recessive and additive inheritance models. Haplotype blocks were identified using HaploView 4.2 software (Broad Institute of MIT and Harvard, Cambridge, MA, USA) [51], using Gabriel et al.’s algorithm [52]. D′ and R^2^ values were used as measures of linkage disequilibrium. The study’s size and power were calculated using Statistica 13.0 software. Statistical significance was acknowledged at *p* < 0.050. Cases with missing data were excluded from the relevant comparisons.

## Figures and Tables

**Table 2:** The distribution of genotype frequencies of the *PDGFRA* gene’s polymorphisms in patients who achieved the minimal clinically important difference threshold in PROM values after PRP therapy (MCID+) and those who did not achieve therapeutic success (MCID–).

Model of Heredity	SNP	Genotype	PROM	Week of Follow-Up	MCID+ Patients		MCID– Patients		*p*
					*n*	%	*n*	%	
Additive	rs6554164	CC	VAS	2	0	0.00	7	10.29	0.004
		CT			18	29.51	29	42.65	
		TT			43	70.49	32	47.06	
		CC	QDASH	104	0	0.00	5	13.89	0.011
		CT			36	41.86	11	30.56	
		TT			50	58.14	20	55.56	
	rs1316926	AA	VAS	12	33	38.37	11	23.91	0.015
		AG			33	38.37	30	65.22	
		GG			20	23.26	5	10.87	
Dominant/recessive	rs7668190	AA	VAS	2	34	55,74	26	38.24	0.039
		AT/TT			27	44.26	42	61.76	
		AA	VAS	8	43	53.75	18	35.29	0.039
		AT/TT			37	46.26	33	64.71	
	rs6554164	CC	VAS	2	0	0.00	7	10.29	0.010
		CT/TT			61	100.00	61	89.71	
		CC	QDASH	2	0	0.00	7	8.43	0.042
		CT/TT			46	100.00	76	91.57	
		CC	QDASH	104	1	1.16	5	13.89	0.009
		CT/TT			85	98.84	31	86.11	

Legend: MCID, minimal clinically important difference; SNP, single nucleotide polymorphism.

**Table 3:** Median (±QD) values of the platelets’ parameters in PRP and whole blood for genotypes of the *PDGFRA* gene’s polymorphisms (dominant/recessive model).

SNP	Platelet Parameter	Median	QD	Median	QD	*p* Mann– Whitney U-test
rs7668190		AA		AT + TT		
	PLT PRP	349.00	90.50	327.00	52.00	0.035
	PCT PRP	0.32	0.08	0.28	0.05	0.042
rs869978		TT		CT + CC		
	PLT	170.50	19.25	246.00	35.00	0.005
	PCT	1.75	0.18	2.32	0.36	0.015
	MPV	10.15	0.10	9.10	0.70	0.043
	PDW	16.30	0.03	16.00	0.15	0.016
rs1316926		AA		AG + GG		
	PLT PRP	307.00	70.75	353.50	58.00	0.032

Legend: SNP, single nucleotide polymorphism; PRP, platelet-rich plasma; QD, quartile deviation.

**Table 4:** The study group’s clinical, demographic and hematological characteristics.

Characteristics			
General	Number of subjects, *N*	107	-
	Number of elbows, *n* (%)	132	(100.0)
	Tennis elbow in the dominant limb, *n* (%)	86	(65.2)
	Age, median ± QD	46.00	5.50
	BMI, median ± QD	25.65	2.00
	Current smokers, *n* (%)	22	(16.6)
Comorbidities	Diabetes mellitus, *n* (%)	4	(3.0)
	Gout, *n* (%)	8	(6.1)
	Thyroid diseases, *n* (%)	15	(11.4)
	Hypertension, *n* (%)	18	(13.6)
Whole Blood parameters	PLT 10^9^/L, median ± QD	240.00	40.50
	PCT mL/L, median ± QD	2.31	0.36
	MPV fl, median ± QD	9.10	0.73
	PDW fl, median ± QD	16.10	0.15
PRP parameters	PLT 10^9^/L, median ± QD	343.00	65.00
	PCT mL/L, median ± QD	0.30	0.06
	MPV fl, median ± QD	8.60	0.40
	PDW fl, median ± QD	14.60	0.25

Legend: BMI, body mass index; PLT, platelets; PCT, plateletcrit; MPV, mean platelet volume; PDW, platelet distribution width; PRP, platelet-rich plasma; QD, quartile deviation.

## Data Availability

Data is contained within the article and Supplementary Materials.

## Supplementary Materials

The following supporting information can be downloaded at https://www.mdpi.com/article/10.3390/ijms25084266/s1.
