# Supplementary material for: Association between Platelet-Derived Growth Factor Receptor Alpha Gene Polymorphisms and Platelet-Rich Plasma’s Efficiency in Treating Lateral Elbow Tendinopathy—A Prospective Cohort Study
Source: Int J Mol Sci. 2024 Apr 12;25(8):4266. doi: 10.3390/ijms25084266 (PMC11050239; doi:10.3390/ijms25084266)
Supplement: Supplementary file 1 [file ijms-25-04266-s001.zip › Supplementary Table 1.docx]

**Table S1.** PROMs values in carriers of different genotypes of the rs7668190 (A>T) polymorphism of the *PDGFRA* gene.

PROMs values in TT homozygotes and carriers of the A allele of the rs7668190 *PDGFRA* gene polymorphism.

| **PROMs** | week | **TT rs7668190** | | **AT+AA rs7668190** | | ***p*** |
| --- | --- | --- | --- | --- | --- | --- |
|  |  | median | ± QD | median | ± QD | **Mann-**  **Whitney**  **U test** |
| VAS | 0 | 4.50 | 1.50 | 6.00 | 2.00 | 0.044* |
|  | 2 | 3.50 | 1.00 | 4.00 | 1.50 | 0.356 |
|  | 4 | 2.50 | 1.00 | 3.00 | 1.50 | 0.573 |
|  | 8 | 2.50 | 2.00 | 3.00 | 2.00 | 0.657 |
|  | 12 | 2.00 | 1.50 | 3.00 | 1.50 | 0.762 |
|  | 24 | 1.00 | 1.50 | 2.00 | 2.00 | 0.336 |
|  | 52 | 1.00 | 2.00 | 2.00 | 2.00 | 0.715 |
|  | 104 | 1.00 | 1.50 | 1.00 | 1.50 | 0.554 |
| ΔVAS (vs week 0) | 2 | 1.00 | 1.00 | 1.00 | 1.50 | 0.237 |
|  | 4 | 1.00 | 1.50 | 2.00 | 1.50 | 0.032* |
|  | 8 | 1.00 | 2.00 | 3.00 | 2.00 | 0.079 |
|  | 12 | 2.00 | 1.50 | 3.00 | 2.00 | 0.294 |
|  | 24 | 2.00 | 1.00 | 3.00 | 2.00 | 0.194 |
|  | 52 | 1.00 | 2.00 | 4.00 | 2.25 | 0.123 |
|  | 104 | 2.00 | 1.50 | 4.00 | 2.50 | 0.027 |
| QDASH | 0 | 50.00 | 14.77 | 52.27 | 13.64 | 0.478 |
|  | 2 | 31.82 | 21.59 | 40.91 | 15.91 | 0.353 |
|  | 4 | 35.23 | 17.05 | 36.36 | 13.64 | 0.923 |
|  | 8 | 25.00 | 14.77 | 34.09 | 19.32 | 0.537 |
|  | 12 | 28.41 | 15.91 | 28.41 | 17.05 | 0.759 |
|  | 24 | 15.91 | 12.50 | 25.00 | 21.59 | 0.367 |
|  | 52 | 18.18 | 11.36 | 18.18 | 23.86 | 0.487 |
|  | 104 | 4.54 | 10.23 | 13.64 | 21.59 | 0.459 |
| ΔQDASH (vs week 0) | 2 | 6.81 | 7.95 | 6.81 | 13.64 | 0.985 |
|  | 4 | 11.36 | 14.77 | 12.50 | 14.77 | 0.490 |
|  | 8 | 13.64 | 20.46 | 15.90 | 18.19 | 0.820 |
|  | 12 | 18.18 | 15.91 | 18.18 | 17.05 | 0.879 |
|  | 24 | 31.81 | 18.18 | 20.45 | 20.32 | 0.849 |
|  | 52 | 18.17 | 15.91 | 22.73 | 21.59 | 0.784 |
|  | 104 | 27.27 | 20.45 | 31.82 | 22.73 | 0.449 |
| PRTEE | 0 | 44.75 | 12.00 | 52.50 | 14.25 | 0.310 |
|  | 2 | 21.50 | 23.00 | 30.50 | 15.75 | 0.406 |
|  | 4 | 22.50 | 14.50 | 25.50 | 13.50 | 0.994 |
|  | 8 | 16.25 | 11.00 | 23.00 | 16.25 | 0.695 |
|  | 12 | 17.00 | 13.75 | 20.75 | 15.00 | 0.668 |
|  | 24 | 9.00 | 15.00 | 15.00 | 17.50 | 0.317 |
|  | 52 | 7.50 | 8.00 | 12.00 | 15.75 | 0.454 |
|  | 104 | 3.00 | 7.75 | 8.00 | 12.75 | 0.515 |
| ΔPRTEE (vs week 0) | 2 | 13.00 | 7.50 | 15.25 | 12.63 | 0.504 |
|  | 4 | 13.00 | 11.00 | 21.75 | 14.00 | 0.137 |
|  | 8 | 24.00 | 12.75 | 28.00 | 15.75 | 0.290 |
|  | 12 | 30.00 | 14.25 | 28.00 | 16.25 | 0.712 |
|  | 24 | 28.00 | 13.25 | 31.00 | 19.25 | 0.589 |
|  | 52 | 29.00 | 12.25 | 33.50 | 18.38 | 0.745 |
|  | 104 | 30.50 | 13.75 | 38.25 | 16.50 | 0.283 |

PROMs values in AA homozygotes and carriers of the T allele of the rs7668190 *PDGFRA* gene polymorphism.

| **PROMs** | week | **AA rs7668190** | | **AT+TT rs7668190** | | ***p*** |
| --- | --- | --- | --- | --- | --- | --- |
|  |  | median | ± QD | median | ± QD | **Mann-**  **Whitney**  **U test** |
| VAS | 0 | 6.00 | 1.75 | 5.00 | 1.50 | 0.127 |
|  | 2 | 4.00 | 1.50 | 4.00 | 2.00 | 0.297 |
|  | 4 | 3.00 | 1.50 | 3.00 | 1.50 | 0.762 |
|  | 8 | 3.00 | 1.50 | 3.00 | 2.50 | 0.379 |
|  | 12 | 3.00 | 1.50 | 2.00 | 2.00 | 0.532 |
|  | 24 | 2.50 | 2.00 | 2.00 | 2.50 | 0.249 |
|  | 52 | 2.00 | 2.50 | 1.00 | 2.00 | 0.504 |
|  | 104 | 1.00 | 1.50 | 1.00 | 1.50 | 0.667 |
| ΔVAS (vs week 0) | 2 | 2.00 | 1.50 | 1.00 | 1.00 | 0.002* |
|  | 4 | 3.00 | 1.50 | 2.00 | 1.50 | 0.054 |
|  | 8 | 3.00 | 2.00 | 2.00 | 2.50 | 0.032* |
|  | 12 | 3.50 | 2.00 | 2.00 | 1.50 | 0.095 |
|  | 24 | 3.00 | 2.00 | 3.00 | 2.00 | 0.994 |
|  | 52 | 3.25 | 2.50 | 4.00 | 2.50 | 0.785 |
|  | 104 | 4.00 | 2.50 | 4.00 | 2.00 | 0.262 |
| QDASH | 0 | 52.27 | 12.37 | 52.27 | 15.46 | 0.904 |
|  | 2 | 38.64 | 13.07 | 43.18 | 18.18 | 0.147 |
|  | 4 | 32.95 | 12.50 | 36.36 | 15.91 | 0.074 |
|  | 8 | 29.55 | 17.05 | 36.36 | 22.73 | 0.289 |
|  | 12 | 25.00 | 15.91 | 31.82 | 20.45 | 0.427 |
|  | 24 | 25.00 | 21.59 | 22.73 | 21.02 | 0.777 |
|  | 52 | 20.45 | 22.73 | 15.91 | 23.86 | 0.613 |
|  | 104 | 13.64 | 20.45 | 11.36 | 21.59 | 0.764 |
| ΔQDASH (vs week 0) | 2 | 11.36 | 16.81 | 2.27 | 11.36 | 0.058 |
|  | 4 | 17.04 | 16.81 | 9.09 | 14.77 | 0.060 |
|  | 8 | 15.91 | 17.04 | 14.77 | 22.73 | 0.270 |
|  | 12 | 18.18 | 17.18 | 18.18 | 20.45 | 0.441 |
|  | 24 | 18.18 | 18.69 | 25.00 | 20.46 | 0.988 |
|  | 52 | 20.31 | 23.93 | 22.72 | 18.18 | 0.594 |
|  | 104 | 31.81 | 22.73 | 29.55 | 22.16 | 0.718 |
| PRTEE | 0 | 52.50 | 12.25 | 52.00 | 14.50 | 0.852 |
|  | 2 | 25.00 | 12.13 | 34.50 | 17.50 | 0.057 |
|  | 4 | 24.25 | 12.13 | 25.50 | 16.50 | 0.373 |
|  | 8 | 22.00 | 10.75 | 22.00 | 21.25 | 0.418 |
|  | 12 | 19.00 | 12.75 | 21.50 | 16.00 | 0.518 |
|  | 24 | 16.50 | 17.63 | 13.75 | 17.13 | 0.501 |
|  | 52 | 16.00 | 16.88 | 10.75 | 12.25 | 0.399 |
|  | 104 | 8.00 | 14.00 | 6.50 | 9.75 | 0.522 |
| ΔPRTEE (vs week 0) | 2 | 18.25 | 13.25 | 13.50 | 8.25 | 0.059 |
|  | 4 | 25.50 | 15.88 | 20.75 | 13.75 | 0.284 |
|  | 8 | 28.00 | 14.25 | 24.75 | 17.75 | 0.334 |
|  | 12 | 26.50 | 16.25 | 29.50 | 15.50 | 0.800 |
|  | 24 | 28.00 | 17.50 | 31.00 | 19.25 | 0.508 |
|  | 52 | 27.00 | 17.75 | 34.00 | 16.50 | 0.152 |
|  | 104 | 37.50 | 15.75 | 38.75 | 19.13 | 0.754 |

Legend: QD, Quartile Deviation; VAS, Visual Analog Scale; QDASH, quick version of Disabilities of the Arm, Shoulder and Hand score; PROM, Patient-Reported Outcome Measures; PRTEE, Patient-Rated Tennis Elbow Evaluation.

*Statistically significant results
