# Supplementary material for: Association between Platelet-Derived Growth Factor Receptor Alpha Gene Polymorphisms and Platelet-Rich Plasma’s Efficiency in Treating Lateral Elbow Tendinopathy—A Prospective Cohort Study
Source: Int J Mol Sci. 2024 Apr 12;25(8):4266. doi: 10.3390/ijms25084266 (PMC11050239; doi:10.3390/ijms25084266)
Supplement: Supplementary file 1 [file ijms-25-04266-s001.zip › Supplementary Table 2.docx]

**Table S2.** PROMs values in carriers of different genotypes of the rs6554164 (T>C) polymorphism of the *PDGFRA* gene.

PROMs values in CC homozygotes and carriers of the T allele of the rs6554164 *PDGFRA* gene polymorphism.

| **PROMs** | week | **CC rs6554164** | | **CT+TT rs6554164** | | ***p*** |
| --- | --- | --- | --- | --- | --- | --- |
|  |  | median | ± QD | median | ± QD | **Mann-**  **Whitney**  **U test** |
| VAS | 0 | 4.00 | 2.00 | 6.00 | 1.88 | 0.110 |
|  | 2 | 4.00 | 1.50 | 4.00 | 1.50 | 0.988 |
|  | 4 | 2.00 | 1.00 | 3.00 | 1.50 | 0.202 |
|  | 8 | 2.00 | 1.50 | 3.00 | 2.00 | 0.399 |
|  | 12 | 2.00 | 2.00 | 3.00 | 1.75 | 0.417 |
|  | 24 | 3.00 | 2.00 | 2.00 | 2.00 | 0.789 |
|  | 52 | 3.00 | 2.50 | 2.00 | 2.00 | 0.797 |
|  | 104 | 1.50 | 1.50 | 1.00 | 1.50 | 0.686 |
| ΔVAS (vs week 0) | 2 | 0.00 | 0.50 | 1.25 | 1.50 | 0.049* |
|  | 4 | 1.00 | 2.00 | 2.00 | 2.00 | 0.245 |
|  | 8 | 2.00 | 1.50 | 2.00 | 2.00 | 0.421 |
|  | 12 | 2.00 | 2.00 | 3.00 | 2.00 | 0.348 |
|  | 24 | 2.00 | 2.50 | 3.00 | 2.00 | 0.077 |
|  | 52 | 0.50 | 1.50 | 4.00 | 2.50 | 0.062 |
|  | 104 | 2.00 | 0.50 | 4.00 | 2.50 | 0.053 |
| QDASH | 0 | 47.72 | 23.86 | 52.27 | 12.50 | 0.206 |
|  | 2 | 36.36 | 19.32 | 40.91 | 15.91 | 0.324 |
|  | 4 | 34.09 | 13.64 | 36.36 | 14.77 | 0.252 |
|  | 8 | 15.91 | 15.91 | 34.09 | 19.32 | 0.381 |
|  | 12 | 29.55 | 21.59 | 28.41 | 17.61 | 0.439 |
|  | 24 | 31.82 | 17.05 | 25.00 | 21.59 | 0.975 |
|  | 52 | 27.27 | 19.32 | 18.18 | 23.86 | 0.719 |
|  | 104 | 18.18 | 21.59 | 13.64 | 20.45 | 0.849 |
| ΔQDASH (vs week 0) | 2 | 6.81 | 6.82 | 6.81 | 13.64 | 0.666 |
|  | 4 | 9.09 | 11.36 | 13.63 | 15.91 | 0.467 |
|  | 8 | 4.54 | 23.86 | 15.91 | 18.29 | 0.560 |
|  | 12 | 2.27 | 31.82 | 20.45 | 15.91 | 0.383 |
|  | 24 | 2.27 | 28.41 | 20.45 | 18.19 | 0.157 |
|  | 52 | 10.23 | 7.95 | 25.00 | 21.59 | 0.125 |
|  | 104 | 7.96 | 5.68 | 32.95 | 22.73 | 0.038* |
| PRTEE | 0 | 38.50 | 13.50 | 52.75 | 14.13 | 0.129 |
|  | 2 | 24.50 | 12.50 | 30.00 | 16.75 | 0.401 |
|  | 4 | 22.00 | 8.25 | 25.50 | 14.50 | 0.461 |
|  | 8 | 9.00 | 13.00 | 24.00 | 15.75 | 0.321 |
|  | 12 | 18.00 | 17.00 | 20.50 | 14.88 | 0.509 |
|  | 24 | 19.50 | 16.00 | 14.75 | 17.13 | 0.899 |
|  | 52 | 17.00 | 9.50 | 11.50 | 15.75 | 0.597 |
|  | 104 | 10.25 | 14.00 | 7.50 | 12.75 | 0.900 |
| ΔPRTEE (vs week 0) | 2 | 14.00 | 7.50 | 15.25 | 12.50 | 0.454 |
|  | 4 | 14.00 | 8.75 | 21.50 | 13.75 | 0.194 |
|  | 8 | 23.50 | 17.00 | 27.50 | 16.75 | 0.356 |
|  | 12 | 13.00 | 22.50 | 29.00 | 16.25 | 0.276 |
|  | 24 | 10.50 | 21.25 | 31.00 | 19.25 | 0.128 |
|  | 52 | 22.25 | 8.25 | 33.50 | 18.50 | 0.215 |
|  | 104 | 20.75 | 7.25 | 38.50 | 16.63 | 0.037* |

PROMs values in TT homozygotes and carriers of the C allele of the rs6554164 *PDGFRA* gene polymorphism.

| **PROMs** | week | **TT rs6554164** | | **CT+CC rs6554164** | | ***p*** |
| --- | --- | --- | --- | --- | --- | --- |
|  |  | median | ± QD | median | ± QD | **Mann-**  **Whitney**  **U test** |
| VAS | 0 | 6.00 | 2.00 | 5.00 | 1.50 | 0.146 |
|  | 2 | 3.00 | 1.50 | 4.00 | 2.00 | 0.185 |
|  | 4 | 3.00 | 1.50 | 4.00 | 1.50 | 0.240 |
|  | 8 | 3.00 | 1.50 | 3.00 | 2.50 | 0.594 |
|  | 12 | 3.00 | 1.50 | 2.00 | 2.00 | 0.586 |
|  | 24 | 2.00 | 2.00 | 2.00 | 2.50 | 0.483 |
|  | 52 | 1.00 | 2.50 | 2.00 | 2.00 | 0.862 |
|  | 104 | 1.00 | 1.50 | 1.00 | 1.50 | 0.378 |
| ΔVAS (vs week 0) | 2 | 2.00 | 1.50 | 1.00 | 1.00 | 0.001* |
|  | 4 | 3.00 | 1.50 | 1.50 | 1.50 | 0.007* |
|  | 8 | 3.00 | 2.50 | 2.00 | 2.00 | 0.060 |
|  | 12 | 3.25 | 2.00 | 2.00 | 1.50 | 0.166 |
|  | 24 | 3.00 | 2.00 | 2.00 | 2.00 | 0.600 |
|  | 52 | 4.00 | 2.50 | 3.00 | 2.50 | 0.470 |
|  | 104 | 4.00 | 2.50 | 3.00 | 2.00 | 0.262 |
| QDASH | 0 | 52.27 | 13.64 | 52.27 | 10.23 | 0.388 |
|  | 2 | 38.64 | 13.64 | 47.73 | 18.18 | 0.093 |
|  | 4 | 34.09 | 12.50 | 43.18 | 17.05 | 0.064 |
|  | 8 | 32.95 | 18.18 | 34.09 | 19.32 | 0.963 |
|  | 12 | 27.27 | 16.48 | 29.55 | 20.45 | 0.889 |
|  | 24 | 25.00 | 21.59 | 26.14 | 22.73 | 0.710 |
|  | 52 | 15.91 | 23.86 | 19.32 | 20.45 | 0.454 |
|  | 104 | 13.64 | 18.18 | 15.91 | 21.59 | 0.909 |
| ΔQDASH (vs week 0) | 2 | 6.81 | 15.91 | 4.55 | 11.37 | 0.454 |
|  | 4 | 15.90 | 15.91 | 11.36 | 13.64 | 0.407 |
|  | 8 | 14.77 | 19.89 | 15.91 | 18.29 | 0.693 |
|  | 12 | 18.18 | 19.39 | 21.59 | 14.78 | 0.642 |
|  | 24 | 18.17 | 19.32 | 27.27 | 19.32 | 0.414 |
|  | 52 | 20.45 | 24.00 | 27.27 | 17.05 | 0.220 |
|  | 104 | 30.68 | 22.73 | 30.68 | 22.15 | 0.675 |
| PRTEE | 0 | 51.00 | 12.75 | 53.00 | 13.75 | 0.391 |
|  | 2 | 27.00 | 11.50 | 36.50 | 20.75 | 0.064 |
|  | 4 | 23.50 | 12.00 | 27.50 | 19.75 | 0.112 |
|  | 8 | 23.00 | 13.50 | 22.00 | 21.25 | 0.825 |
|  | 12 | 20.00 | 14.63 | 21.00 | 18.00 | 0.666 |
|  | 24 | 15.00 | 17.00 | 14.50 | 17.63 | 0.627 |
|  | 52 | 12.00 | 16.25 | 11.75 | 12.50 | 0.575 |
|  | 104 | 7.75 | 12.75 | 7.00 | 12.50 | 0.921 |
| ΔPRTEE (vs week 0) | 2 | 15.00 | 13.25 | 15.00 | 9.50 | 0.257 |
|  | 4 | 26.00 | 14.25 | 20.25 | 12.75 | 0.225 |
|  | 8 | 25.25 | 15.75 | 26.75 | 16.75 | 0.962 |
|  | 12 | 25.25 | 17.75 | 29.25 | 13.50 | 0.822 |
|  | 24 | 27.00 | 19.50 | 32.50 | 18.50 | 0.386 |
|  | 52 | 27.00 | 18.75 | 35.00 | 16.25 | 0.155 |
|  | 104 | 37.50 | 16.75 | 39.00 | 14.75 | 0.639 |

Legend: QD, Quartile Deviation; VAS, Visual Analog Scale; QDASH, quick version of Disabilities of the Arm, Shoulder and Hand score; PROM, Patient-Reported Outcome Measures; PRTEE, Patient-Rated Tennis Elbow Evaluation.

*Statistically significant results
