# Supplementary material for: Association between Platelet-Derived Growth Factor Receptor Alpha Gene Polymorphisms and Platelet-Rich Plasma’s Efficiency in Treating Lateral Elbow Tendinopathy—A Prospective Cohort Study
Source: Int J Mol Sci. 2024 Apr 12;25(8):4266. doi: 10.3390/ijms25084266 (PMC11050239; doi:10.3390/ijms25084266)
Supplement: Supplementary file 1 [file ijms-25-04266-s001.zip › Supplementary Table 3.docx]

**Table S3.** PROMs values in carriers of different genotypes of the rs869978 (T>C) polymorphism of the *PDGFRA* gene.

PROMs values in TT homozygotes and carriers of the C allele of the rs869978 *PDGFRA* gene polymorphism.

| **PROMs** | week | **TT rs869978** | | **CT+CC rs869978** | | ***p*** |
| --- | --- | --- | --- | --- | --- | --- |
|  |  | median | ± QD | median | ± QD | **Mann-**  **Whitney**  **U test** |
| VAS | 0 | 4.00 | 3.00 | 6.00 | 1.50 | 0.631 |
|  | 2 | 1.00 | 0.50 | 4.00 | 1.50 | 0.021* |
|  | 4 | 2.00 | 1.50 | 3.00 | 1.50 | 0.240 |
|  | 8 | 3.00 | 1.50 | 3.00 | 2.00 | 0.455 |
|  | 12 | 3.00 | 1.00 | 2.00 | 2.00 | 0.584 |
|  | 24 | 2.00 | 1.00 | 2.00 | 2.00 | 0.741 |
|  | 52 | 0.00 | 1.50 | 2.00 | 2.00 | 0.298 |
|  | 104 | 1.00 | 1.00 | 1.00 | 1.50 | 0.745 |
| ΔVAS (vs week 0) | 2 | 3.00 | 2.50 | 1.00 | 1.50 | 0.278 |
|  | 4 | 3.00 | 2.00 | 2.00 | 2.00 | 0.802 |
|  | 8 | 3.00 | 1.50 | 2.00 | 2.00 | 0.650 |
|  | 12 | 3.00 | 1.50 | 3.00 | 2.00 | 0.763 |
|  | 24 | 3.00 | 1.50 | 3.00 | 2.00 | 0.916 |
|  | 52 | 4.00 | 1.50 | 3.25 | 2.00 | 0.699 |
|  | 104 | 3.00 | 2.00 | 4.00 | 2.00 | 0.871 |
| QDASH | 0 | 59.09 | 25.00 | 52.27 | 13.64 | 0.404 |
|  | 2 | 20.45 | 15.91 | 39.77 | 15.91 | 0.261 |
|  | 4 | 20.45 | 18.18 | 36.36 | 13.64 | 0.370 |
|  | 8 | 22.73 | 15.91 | 34.09 | 19.32 | 0.213 |
|  | 12 | 31.82 | 7.95 | 27.27 | 18.18 | 0.629 |
|  | 24 | 20.45 | 15.91 | 25.00 | 21.59 | 0.355 |
|  | 52 | 0.00 | 6.82 | 20.45 | 22.73 | 0.101 |
|  | 104 | 20.45 | 10.23 | 13.64 | 21.59 | 0.676 |
| ΔQDASH (vs week 0) | 2 | 4.55 | 12.50 | 6.81 | 13.63 | 0.744 |
|  | 4 | 0.01 | 10.23 | 13.63 | 14.77 | 0.498 |
|  | 8 | 6.82 | 18.18 | 15.91 | 18.29 | 0.904 |
|  | 12 | 20.45 | 12.50 | 18.18 | 17.05 | 0.741 |
|  | 24 | 13.64 | 18.18 | 20.45 | 19.19 | 0.819 |
|  | 52 | 18.18 | 18.18 | 22.72 | 21.59 | 0.864 |
|  | 104 | 15.91 | 14.77 | 30.68 | 22.73 | 0.450 |
| PRTEE | 0 | 40.00 | 26.50 | 52.50 | 13.25 | 0.784 |
|  | 2 | 13.00 | 8.00 | 30.25 | 16.00 | 0.205 |
|  | 4 | 8.00 | 12.75 | 24.75 | 14.25 | 0.254 |
|  | 8 | 20.00 | 9.75 | 24.00 | 16.25 | 0.262 |
|  | 12 | 24.00 | 9.25 | 20.00 | 15.50 | 0.567 |
|  | 24 | 13.50 | 9.75 | 15.00 | 17.75 | 0.324 |
|  | 52 | 0.00 | 4.75 | 12.50 | 15.50 | 0.110 |
|  | 104 | 6.50 | 3.75 | 7.50 | 14.00 | 0.465 |
| ΔPRTEE (vs week 0) | 2 | 14.00 | 13.75 | 15.00 | 11.25 | 0.947 |
|  | 4 | 32.50 | 11.00 | 21.50 | 13.00 | 0.460 |
|  | 8 | 40.00 | 9.00 | 25.50 | 16.75 | 0.414 |
|  | 12 | 34.00 | 14.50 | 28.50 | 16.25 | 0.501 |
|  | 24 | 40.00 | 11.75 | 30.50 | 18.00 | 0.503 |
|  | 52 | 40.00 | 12.75 | 32.75 | 18.00 | 0.407 |
|  | 104 | 33.50 | 14.25 | 38.00 | 16.25 | 0.828 |

PROMs values in CC homozygotes and carriers of the T allele of the rs869978 *PDGFRA* gene polymorphism.

| **PROMs** | week | **CC rs869978** | | **CT+TT rs869978** | | ***p*** |
| --- | --- | --- | --- | --- | --- | --- |
|  |  | median | ± QD | median | ± QD | **Mann-**  **Whitney**  **U test** |
| VAS | 0 | 6.00 | 1.75 | 6.00 | 1.50 | 0.708 |
|  | 2 | 4.00 | 1.50 | 4.00 | 2.00 | 0.778 |
|  | 4 | 3.00 | 1.50 | 4.00 | 2.00 | 0.709 |
|  | 8 | 3.00 | 2.00 | 3.00 | 2.50 | 0.370 |
|  | 12 | 3.00 | 1.50 | 2.00 | 2.00 | 0.400 |
|  | 24 | 2.00 | 2.00 | 2.00 | 2.50 | 0.552 |
|  | 52 | 1.50 | 2.50 | 2.00 | 2.00 | 0.528 |
|  | 104 | 1.00 | 1.50 | 1.00 | 1.50 | 0.684 |
| ΔVAS (vs week 0) | 2 | 1.00 | 1.50 | 1.00 | 1.50 | 0.244 |
|  | 4 | 2.00 | 1.50 | 2.00 | 2.00 | 0.374 |
|  | 8 | 2.00 | 2.00 | 3.00 | 2.00 | 0.666 |
|  | 12 | 2.00 | 2.00 | 3.00 | 2.00 | 0.364 |
|  | 24 | 2.00 | 2.00 | 3.00 | 2.00 | 0.566 |
|  | 52 | 3.00 | 2.00 | 4.00 | 2.50 | 0.735 |
|  | 104 | 4.00 | 2.50 | 4.00 | 2.00 | 0.685 |
| QDASH | 0 | 52.27 | 13.64 | 56.81 | 10.23 | 0.340 |
|  | 2 | 38.64 | 13.07 | 40.91 | 22.73 | 0.735 |
|  | 4 | 36.36 | 12.50 | 36.36 | 18.18 | 0.503 |
|  | 8 | 34.09 | 17.05 | 29.55 | 20.45 | 0.392 |
|  | 12 | 29.55 | 17.05 | 27.27 | 21.59 | 0.596 |
|  | 24 | 25.00 | 21.59 | 20.45 | 22.73 | 0.745 |
|  | 52 | 17.05 | 23.86 | 20.45 | 19.32 | 0.600 |
|  | 104 | 13.64 | 17.05 | 9.09 | 22.73 | 0.938 |
| ΔQDASH (vs week 0) | 2 | 5.68 | 14.21 | 6.82 | 12.50 | 0.603 |
|  | 4 | 11.36 | 15.34 | 13.64 | 15.91 | 0.765 |
|  | 8 | 13.63 | 16.02 | 24.54 | 18.18 | 0.219 |
|  | 12 | 15.97 | 17.05 | 27.27 | 17.18 | 0.213 |
|  | 24 | 18.18 | 19.32 | 27.27 | 22.09 | 0.480 |
|  | 52 | 20.45 | 23.86 | 27.27 | 15.91 | 0.217 |
|  | 104 | 29.54 | 22.73 | 31.82 | 22.66 | 0.976 |
| PRTEE | 0 | 50.00 | 12.50 | 53.50 | 13.75 | 0.299 |
|  | 2 | 29.75 | 14.00 | 27.50 | 19.50 | 0.585 |
|  | 4 | 24.75 | 11.88 | 24.50 | 23.25 | 0.629 |
|  | 8 | 26.50 | 13.50 | 20.00 | 20.25 | 0.316 |
|  | 12 | 20.00 | 14.25 | 18.00 | 19.75 | 0.712 |
|  | 24 | 15.50 | 16.50 | 13.50 | 18.25 | 0.607 |
|  | 52 | 11.50 | 16.25 | 12.75 | 11.50 | 0.652 |
|  | 104 | 8.00 | 12.75 | 5.50 | 12.75 | 0.812 |
| ΔPRTEE (vs week 0) | 2 | 14.00 | 11.63 | 16.75 | 13.25 | 0.756 |
|  | 4 | 21.50 | 12.38 | 21.50 | 17.50 | 0.948 |
|  | 8 | 25.00 | 14.00 | 30.25 | 20.00 | 0.161 |
|  | 12 | 24.00 | 16.50 | 30.75 | 16.75 | 0.202 |
|  | 24 | 28.50 | 18.25 | 31.75 | 18.00 | 0.376 |
|  | 52 | 30.25 | 18.50 | 35.00 | 16.25 | 0.175 |
|  | 104 | 37.50 | 17.25 | 39.70 | 14.38 | 0.593 |

Legend: QD, Quartile Deviation; VAS, Visual Analog Scale; QDASH, quick version of Disabilities of the Arm, Shoulder and Hand score; PROM, Patient-Reported Outcome Measures; PRTEE, Patient-Rated Tennis Elbow Evaluation.

*Statistically significant results
