# Supplementary material for: Association between Platelet-Derived Growth Factor Receptor Alpha Gene Polymorphisms and Platelet-Rich Plasma’s Efficiency in Treating Lateral Elbow Tendinopathy—A Prospective Cohort Study
Source: Int J Mol Sci. 2024 Apr 12;25(8):4266. doi: 10.3390/ijms25084266 (PMC11050239; doi:10.3390/ijms25084266)
Supplement: Supplementary file 1 [file ijms-25-04266-s001.zip › Supplementary Table 4.docx]

**Table S4.** PROMs values in carriers of different genotypes of the rs1316926 (G>A) polymorphism of the *PDGFRA* gene.

PROMs values in GG homozygotes and carriers of the A allele of the rs1316926 *PDGFRA* gene polymorphism.

| **PROMs** | week | **GG rs1316926** | | **AG+AA rs1316926** | | ***p*** |
| --- | --- | --- | --- | --- | --- | --- |
|  |  | median | ± QD | median | ± QD | **Mann-**  **Whitney**  **U test** |
| VAS | 0 | 6.00 | 2.00 | 6.00 | 1.50 | 0.308 |
|  | 2 | 4.00 | 1.50 | 4.00 | 1.50 | 0.638 |
|  | 4 | 3.00 | 3.00 | 3.00 | 1.50 | 0.751 |
|  | 8 | 4.00 | 2.25 | 3.00 | 2.00 | 0.723 |
|  | 12 | 2.50 | 2.00 | 2.50 | 2.00 | 0.360 |
|  | 24 | 1.00 | 2.00 | 2.00 | 2.00 | 0.145 |
|  | 52 | 1.50 | 2.00 | 2.00 | 2.50 | 0.566 |
|  | 104 | 1.00 | 1.50 | 1.00 | 1.50 | 0.744 |
| ΔVAS (vs week 0) | 2 | 1.00 | 1.50 | 1.00 | 1.50 | 0.863 |
|  | 4 | 2.50 | 2.00 | 2.00 | 2.00 | 0.827 |
|  | 8 | 3.00 | 1.88 | 2.00 | 2.00 | 0.797 |
|  | 12 | 4.00 | 1.63 | 2.00 | 2.00 | 0.132 |
|  | 24 | 4.00 | 1.88 | 2.00 | 2.00 | 0.068 |
|  | 52 | 4.50 | 2.50 | 3.00 | 2.00 | 0.160 |
|  | 104 | 5.00 | 2.13 | 4.00 | 2.00 | 0.263 |
| QDASH | 0 | 56.81 | 15.34 | 52.27 | 13.64 | 0.104 |
|  | 2 | 40.91 | 19.32 | 38.64 | 15.34 | 0.208 |
|  | 4 | 40.91 | 15.91 | 35.23 | 14.20 | 0.147 |
|  | 8 | 32.95 | 21.59 | 32.95 | 18.75 | 0.551 |
|  | 12 | 23.86 | 25.00 | 29.55 | 17.05 | 0.281 |
|  | 24 | 12.50 | 22.16 | 26.14 | 21.59 | 0.569 |
|  | 52 | 14.77 | 24.43 | 18.18 | 21.59 | 0.695 |
|  | 104 | 14.78 | 21.02 | 12.50 | 21.02 | 0.758 |
| ΔQDASH (vs week 0) | 2 | 2.27 | 12.50 | 6.81 | 13.64 | 0.494 |
|  | 4 | 13.64 | 9.09 | 11.36 | 16.91 | 0.774 |
|  | 8 | 13.63 | 15.91 | 15.91 | 20.45 | 0.601 |
|  | 12 | 23.86 | 14.77 | 15.97 | 18.18 | 0.085 |
|  | 24 | 29.54 | 16.48 | 18.17 | 20.45 | 0.130 |
|  | 52 | 27.27 | 17.05 | 20.45 | 22.73 | 0.234 |
|  | 104 | 34.09 | 20.46 | 29.55 | 22.73 | 0.382 |
| PRTEE | 0 | 50.75 | 12.88 | 52.50 | 13.75 | 0.754 |
|  | 2 | 36.00 | 18.00 | 28.00 | 15.38 | 0.236 |
|  | 4 | 24.50 | 16.25 | 24.75 | 13.13 | 0.384 |
|  | 8 | 21.50 | 20.50 | 23.00 | 13.75 | 0.622 |
|  | 12 | 16.25 | 18.13 | 21.25 | 14.38 | 0.307 |
|  | 24 | 10.00 | 17.38 | 15.75 | 16.63 | 0.387 |
|  | 52 | 9.50 | 14.75 | 11.75 | 15.50 | 0.409 |
|  | 104 | 7.25 | 13.25 | 7.25 | 12.75 | 0.731 |
| ΔPRTEE (vs week 0) | 2 | 13.00 | 8.75 | 16.50 | 13.25 | 0.193 |
|  | 4 | 22.00 | 12.75 | 21.00 | 13.75 | 0.728 |
|  | 8 | 26.50 | 14.63 | 25.50 | 15.95 | 0.494 |
|  | 12 | 30.25 | 10.88 | 28.00 | 16.50 | 0.312 |
|  | 24 | 36.25 | 15.63 | 28.50 | 18.25 | 0.337 |
|  | 52 | 35.25 | 15.38 | 32.00 | 17.50 | 0.277 |
|  | 104 | 39.25 | 15.38 | 38.00 | 16.50 | 0.683 |

PROMs values in AA homozygotes and carriers of the G allele of the rs1316926 *PDGFRA* gene polymorphism.

| **PROMs** | week | **AA rs1316926** | | **AG+GG rs1316926** | | ***p*** |
| --- | --- | --- | --- | --- | --- | --- |
|  |  | median | ± QD | median | ± QD | **Mann-**  **Whitney**  **U test** |
| VAS | 0 | 5.50 | 1.50 | 6.00 | 2.00 | 0.305 |
|  | 2 | 3.00 | 1.50 | 4.00 | 1.50 | 0.218 |
|  | 4 | 2.50 | 1.00 | 3.00 | 1.50 | 0.087 |
|  | 8 | 2.00 | 1.50 | 3.50 | 1.50 | 0.017 |
|  | 12 | 1.50 | 1.50 | 3.00 | 2.00 | 0.111 |
|  | 24 | 1.50 | 2.50 | 2.00 | 2.00 | 0.424 |
|  | 52 | 1.00 | 2.50 | 2.00 | 2.00 | 0.415 |
|  | 104 | 1.00 | 1.50 | 1.00 | 1.50 | 0.844 |
| ΔVAS (vs week 0) | 2 | 1.50 | 1.50 | 1.00 | 1.50 | 0.748 |
|  | 4 | 2.00 | 1.50 | 2.00 | 2.00 | 0.477 |
|  | 8 | 3.00 | 1.50 | 2.00 | 2.00 | 0.210 |
|  | 12 | 2.00 | 1.75 | 3.00 | 2.50 | 0.464 |
|  | 24 | 3.00 | 2.00 | 3.00 | 2.00 | 0.875 |
|  | 52 | 3.00 | 2.00 | 4.00 | 2.50 | 0.679 |
|  | 104 | 3.00 | 1.50 | 4.25 | 2.50 | 0.145 |
| QDASH | 0 | 48.86 | 13.00 | 52.27 | 14.77 | 0.308 |
|  | 2 | 32.95 | 15.91 | 40.91 | 15.91 | 0.265 |
|  | 4 | 31.82 | 15.91 | 38.64 | 13.64 | 0.042* |
|  | 8 | 28.41 | 17.61 | 35.23 | 18.75 | 0.026* |
|  | 12 | 26.14 | 18.75 | 29.55 | 17.61 | 0.188 |
|  | 24 | 27.27 | 23.86 | 25.00 | 19.32 | 0.727 |
|  | 52 | 13.64 | 22.73 | 20.45 | 23.86 | 0.913 |
|  | 104 | 13.64 | 20.46 | 13.64 | 20.45 | 0.617 |
| ΔQDASH (vs week 0) | 2 | 4.55 | 14.77 | 6.81 | 12.50 | 0.883 |
|  | 4 | 16.59 | 14.26 | 11.36 | 15.91 | 0.393 |
|  | 8 | 18.18 | 18.81 | 11.36 | 19.32 | 0.284 |
|  | 12 | 18.18 | 20.46 | 18.18 | 15.91 | 0.796 |
|  | 24 | 15.91 | 20.45 | 20.45 | 19.32 | 0.507 |
|  | 52 | 20.45 | 21.59 | 25.00 | 21.59 | 0.612 |
|  | 104 | 29.54 | 20.46 | 34.09 | 20.46 | 0.164 |
| PRTEE | 0 | 47.25 | 12.38 | 53.75 | 14.38 | 0.240 |
|  | 2 | 24.75 | 16.25 | 33.00 | 16.50 | 0.171 |
|  | 4 | 20.75 | 13.88 | 27.50 | 14.25 | 0.075 |
|  | 8 | 16.75 | 12.25 | 26.75 | 16.00 | 0.012* |
|  | 12 | 15.00 | 14.13 | 21.50 | 17.63 | 0.081 |
|  | 24 | 11.50 | 18.00 | 16.50 | 16.50 | 0.462 |
|  | 52 | 5.50 | 17.50 | 13.00 | 14.75 | 0.674 |
|  | 104 | 7.50 | 14.75 | 7.00 | 12.75 | 0.691 |
| ΔPRTEE (vs week 0) | 2 | 17.75 | 11.00 | 14.00 | 11.50 | 0.571 |
|  | 4 | 22.75 | 12.50 | 20.75 | 13.50 | 0.832 |
|  | 8 | 29.50 | 14.60 | 25.00 | 17.50 | 0.303 |
|  | 12 | 28.00 | 13.23 | 29.50 | 17.50 | 0.739 |
|  | 24 | 27.70 | 14.50 | 31.00 | 19.25 | 0.537 |
|  | 52 | 27.00 | 15.75 | 35.25 | 17.50 | 0.249 |
|  | 104 | 31.50 | 14.75 | 38.75 | 15.50 | 0.116 |

Legend: QD, Quartile Deviation; VAS, Visual Analog Scale; QDASH, quick version of Disabilities of the Arm, Shoulder and Hand score; PROM, Patient-Reported Outcome Measures; PRTEE, Patient-Rated Tennis Elbow Evaluation.

*Statistically significant results
