# Supplementary material for: Association between Platelet-Derived Growth Factor Receptor Alpha Gene Polymorphisms and Platelet-Rich Plasma’s Efficiency in Treating Lateral Elbow Tendinopathy—A Prospective Cohort Study
Source: Int J Mol Sci. 2024 Apr 12;25(8):4266. doi: 10.3390/ijms25084266 (PMC11050239; doi:10.3390/ijms25084266)
Supplement: Supplementary file 1 [file ijms-25-04266-s001.zip › Supplementary Table 5.docx]

**Table S5.** Median (±QD) of platelet parameters values in whole blood (WB) and platelet-rich plasma (PRP) for genotypes of the *PDGFRA* gene polymorphisms in recessive/dominant model.

| **Parameter** | **Source** | | **rs7668190 (A>T)** | | | | ***P*. Mann-Whitney**  **U test** |
| --- | --- | --- | --- | --- | --- | --- | --- |
|  |  |  | **TT** | | **AT+AA** | |  |
|  |  |  | **Median** | **±QD** | **Median** | **±QD** |  |
| PLT 10^9^/l | WB | | 229.00 | 59.00 | 242.00 | 32.50 | 0.735 |
| PCT ml/l | WB | | 2.33 | 0.56 | 2.31 | 0.34 | 0.895 |
| MPV fl | WB | | 9.50 | 0.65 | 9.10 | 0.80 | 0.966 |
| PDW fl | WB | | 16.20 | 0.35 | 16.10 | 0.15 | 0.658 |
| PLT 10^9^/l | PRP | | 286.50 | 71.00 | 349.00 | 70.50 | 0.047* |
| PCT ml/l | PRP | | 0.25 | 0.06 | 0.31 | 0.05 | 0.041* |
| MPV fl | PRP | | 8.35 | 0.20 | 8.60 | 0.45 | 0.142 |
| PDW fl | PRP | | 14.35 | 0.25 | 14.60 | 0.23 | 0.183 |

| **Parameter** | **Source** | | **rs7668190 (A>T)** | | | | ***P*. Mann-Whitney**  **U test** |
| --- | --- | --- | --- | --- | --- | --- | --- |
|  |  |  | **AA** | | **AT+TT** | |  |
|  |  |  | **Median** | **±QD** | **Median** | **±QD** |  |
| PLT 10^9^/l | WB | | 244.00 | 39.00 | 229.00 | 34.50 | 0.373 |
| PCT ml/l | WB | | 2.33 | 0.35 | 2.25 | 0.34 | 0.561 |
| MPV fl | WB | | 9.00 | 0.70 | 9.40 | 0.80 | 0.376 |
| PDW fl | WB | | 16.00 | 0.12 | 16.10 | 0.20 | 0.080 |
| PLT 10^9^/l | PRP | | 349.00 | 90.50 | 327.00 | 52.00 | 0.035* |
| PCT ml/l | PRP | | 0.32 | 0.08 | 0.28 | 0.05 | 0.042* |
| MPV fl | PRP | | 8.80 | 0.50 | 8.50 | 0.35 | 0.064 |
| PDW fl | PRP | | 14.60 | 0.20 | 14.50 | 0.20 | 0.162 |

| **Parameter** | **Source** | | **rs6554164 (T>C)** | | | | ***P*. Mann-Whitney**  **U test** |
| --- | --- | --- | --- | --- | --- | --- | --- |
|  |  |  | **CC** | | **CT+TT** | |  |
|  |  |  | **Median** | **±QD** | **Median** | **±QD** |  |
| PLT 10^9^/l | WB | | 188.00 | 58.50 | 244.00 | 35.00 | 0.108 |
| PCT ml/l | WB | | 1.80 | 0.37 | 2.32 | 0.36 | 0.069 |
| MPV fl | WB | | 9.60 | 0.80 | 9.10 | 0.73 | 0.562 |
| PDW fl | WB | | 16.40 | 0.20 | 16.00 | 0.15 | 0.070 |
| PLT 10^9^/l | PRP | | 269.00 | 76.50 | 347.00 | 63.50 | 0.194 |
| PCT ml/l | PRP | | 0.22 | 0.06 | 0.30 | 0.05 | 0.194 |
| MPV fl | PRP | | 8.40 | 0.15 | 8.60 | 0.45 | 0.384 |
| PDW fl | PRP | | 14.30 | 0.15 | 14.60 | 0.25 | 0.104 |

| **Parameter** | **Source** | | **rs6554164 (T>C)** | | | | ***P*. Mann-Whitney**  **U test** |
| --- | --- | --- | --- | --- | --- | --- | --- |
|  |  |  | **TT** | | **CT+CC** | |  |
|  |  |  | **Median** | **±QD** | **Median** | **±QD** |  |
| PLT 10^9^/l | WB | | 230.00 | 37.00 | 253.00 | 48.50 | 0.850 |
| PCT ml/l | WB | | 2.32 | 0.29 | 2.31 | 0.41 | 0.997 |
| MPV fl | WB | | 9.10 | 0.73 | 9.40 | 0.75 | 0.977 |
| PDW fl | WB | | 16.10 | 0.15 | 16.00 | 0.20 | 0.865 |
| PLT 10^9^/l | PRP | | 347.00 | 70.00 | 326.00 | 60.50 | 0.130 |
| PCT ml/l | PRP | | 0.30 | 0.05 | 0.30 | 0.06 | 0.160 |
| MPV fl | PRP | | 8.70 | 0.50 | 8.50 | 0.30 | 0.190 |
| PDW fl | PRP | | 14.60 | 0.25 | 14.50 | 0.20 | 0.238 |

| **Parameter** | **Source** | | **rs869978 (T>C)** | | | | ***P*. Mann-Whitney**  **U test** |
| --- | --- | --- | --- | --- | --- | --- | --- |
|  |  |  | **TT** | | **CT+CC** | |  |
|  |  |  | **Median** | **±QD** | **Median** | **±QD** |  |
| PLT 10^9^/l | WB | | 170.50 | 19.25 | 246.00 | 35.00 | 0.005* |
| PCT ml/l | WB | | 1.75 | 0.18 | 2.32 | 0.36 | 0.015* |
| MPV fl | WB | | 10.15 | 0.10 | 9.10 | 0.70 | 0.043* |
| PDW fl | WB | | 16.30 | 0.03 | 16.00 | 0.15 | 0.016* |
| PLT 10^9^/l | PRP | | 353.00 | 64.50 | 341.00 | 65.00 | 0.370 |
| PCT ml/l | PRP | | 0.28 | 0.07 | 0.30 | 0.06 | 0.390 |
| MPV fl | PRP | | 8.50 | 0.45 | 8.60 | 0.40 | 1.000 |
| PDW fl | PRP | | 14.40 | 0.20 | 14.60 | 0.25 | 0.394 |

| **Parameter** | **Source** | | **rs869978 (T>C)** | | | | ***P*. Mann-Whitney**  **U test** |
| --- | --- | --- | --- | --- | --- | --- | --- |
|  |  |  | **CC** | | **CT+TT** | |  |
|  |  |  | **Median** | **±QD** | **Median** | **±QD** |  |
| PLT 10^9^/l | WB | | 244.00 | 31.75 | 230.00 | 50.00 | 0.674 |
| PCT ml/l | WB | | 2.32 | 0.30 | 2.11 | 0.44 | 0.504 |
| MPV fl | WB | | 9.05 | 0.70 | 9.45 | 0.77 | 0.812 |
| PDW fl | WB | | 16.05 | 0.15 | 16.10 | 0.25 | 0.969 |
| PLT 10^9^/l | PRP | | 349.00 | 88.00 | 328.00 | 52.50 | 0.075 |
| PCT ml/l | PRP | | 0.29 | 0.06 | 0.30 | 0.05 | 0.184 |
| MPV fl | PRP | | 8.50 | 0.45 | 8.60 | 0.40 | 0.851 |
| PDW fl | PRP | | 14.60 | 0.25 | 14.50 | 0.20 | 0.339 |

| **Parameter** | **Source** | | **rs1316926 (G>A)** | | | | ***P*. Mann-Whitney**  **U test** |
| --- | --- | --- | --- | --- | --- | --- | --- |
|  |  |  | **GG** | | **AG+AA** | |  |
|  |  |  | **Median** | **±QD** | **Median** | **±QD** |  |
| PLT 10^9^/l | WB | | 242.00 | 21.75 | 240.00 | 41.00 | 0.807 |
| PCT ml/l | WB | | 2.23 | 0.35 | 2.32 | 0.36 | 0.764 |
| MPV fl | WB | | 9.25 | 0.63 | 9.10 | 0.80 | 0.997 |
| PDW fl | WB | | 16.15 | 0.20 | 16.05 | 0.15 | 0.286 |
| PLT 10^9^/l | PRP | | 360.00 | 58.75 | 336.50 | 65.00 | 0.540 |
| PCT ml/l | PRP | | 0.31 | 0.05 | 0.29 | 0.06 | 0.986 |
| MPV fl | PRP | | 8.55 | 0.35 | 8.60 | 0.45 | 0.271 |
| PDW fl | PRP | | 14.50 | 0.20 | 14.60 | 0.25 | 0.163 |

| **Parameter** | **Source** | | **rs1316926 (G>A)** | | | | ***P*. Mann-Whitney**  **U test** |
| --- | --- | --- | --- | --- | --- | --- | --- |
|  |  |  | **AA** | | **AG+GG** | |  |
|  |  |  | **Median** | **±QD** | **Median** | **±QD** |  |
| PLT 10^9^/l | WB | | 233.50 | 55.00 | 246.00 | 29.50 | 0.414 |
| PCT ml/l | WB | | 2.15 | 0.46 | 2.32 | 0.37 | 0.396 |
| MPV fl | WB | | 9.20 | 0.80 | 9.10 | 0.75 | 0.575 |
| PDW fl | WB | | 16.00 | 0.20 | 16.10 | 0.15 | 0.368 |
| PLT 10^9^/l | PRP | | 307.00 | 70.75 | 353.50 | 58.00 | 0.032* |
| PCT ml/l | PRP | | 0.27 | 0.07 | 0.31 | 0.05 | 0.137 |
| MPV fl | PRP | | 8.50 | 0.48 | 8.60 | 0.40 | 0.855 |
| PDW fl | PRP | | 14.50 | 0.20 | 14.60 | 0.20 | 0.663 |

Legend: MPV, platelet volume; PCT, plateletcrit; *PDGFRA*, platelet-derived growth factor receptor alpha gene; PDW, platelet distribution width; PLT, platelets; QD, Quartile Deviation; WBC, white blood cells; RBC, red blood cells; EOS, eosinophils.

* Statistically significant results
